# Incidence and survival of rare cancers in the US and Europe

**DOI:** 10.1002/cam4.3137

**Published:** 2020-05-21

**Authors:** Laura Botta, Gemma Gatta, Annalisa Trama, Alice Bernasconi, Elad Sharon, Riccardo Capocaccia, Angela B. Mariotto, the RARECAREnet working group

**Affiliations:** ^1^ Evaluative Epidemiology Unit Fondazione IRCCS Istituto Nazionale Tumori Milan Italy; ^2^ Cancer Therapy Evaluation Program National Cancer Institute Bethesda MD USA; ^3^ Editorial Board Epidemiologia & Prevenzione Castellanza Varese Italy; ^4^ Division of Cancer Control and Population Sciences National Cancer Institute Bethesda MD USA

**Keywords:** 5 years net survival, differences, Europe, incidence, rare cancers, USA

## Abstract

Geographical variability of cancer burden was almost exclusively estimated for common cancers. Since rare cancers (RC) have become an area of priority for basic and clinical research and public health organizations, this paper provides, using a common methodology, a detailed comparison of incidence and survival for RC in the US and Europe. We estimated incidence and net survival of 199 malignant RC from data of 2 580 000 patients collected by 18 US‐SEER and 94 European registries, diagnosed within the most recent common period 2000‐2007. RC were defined according to the criterion of crude annual incidence rates <6/100 000. In total, 196 RC were classified as rare in both populations. Of these, 43 had incidence rates significantly different by at least 0.2 per 100 000:34 higher in the US and 9 higher in Europe. Five‐year net survival for all RC combined significantly differed: 54% in the US and 48% in Europe. Survival for 62 RC was significantly higher in the US vs 6 higher in Europe. Differences were not concentrated in a particular cancer family, and were mostly relevant for cases diagnosed >65+ years of age. Use of standardized methods evidenced that incidence and survival rate of majority of RC were higher in the United States compared to Europe. Possible reasons for such differences, requiring further studies, include distribution of risk factors, ability to diagnose RC, different registration practices, and use of updated International Classification of Diseases for Oncology.

## Graphical abstract

Plot of crude incidence rates of rare and common cancers in Europe (y‐axis) vs the US (x‐axis) shows that most cancers were rare (crude incidence <6/100.000, low left square) in both population. Two cancers (*thyroid carcinoma* and *Diffuse B lymphoma*) classified as rare in the European population, however, were common in the US (low right square). In contrast, *breast invasive lobular carcinoma* was common in Europe and rare in the US (high left square).

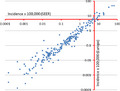

## 1 INTRODUCTION

Patients diagnosed with rate cancers (RC) are disadvantaged with respect to those diagnosed with common cancers. Due to their rarity, diagnosis of RC is often delayed, clinical research is difficult, clinical management is often complex and hard outside a few specialized centers. Two recent papers described the burden of RC in the US^1^ and Europe^2^ and estimated that a significant proportion, 20%‐24% of all patients diagnosed with cancer, are rare. Both papers defined rarity using an annual incidence rate cut‐off <6/100 000, and they used the same list of clinically relevant and histologically defined RC. The list was developed by the Surveillance of RC in Europe (RARECARE) group, which consists of pathologists, hematologists, other clinicians, and epidemiologists. Inferior clinical outcomes of rare vs common cancers were reported in both papers, partially explained by the differential distribution of stage at diagnosis between rare and common cancers as reported in the US.^1^ The European paper reported a low level of centralization for RC patients treatment.^2^

Rare cancers have become an area of priority for basic and clinical research, and public health organizations. The most relevant initiatives in Europe are the European Community‐supported Joint Action on RC (JARC),^3^ whose major goal is to include RC in national cancer plans, and the development of the European Reference Network (ERN)^4^ on RC, aimed at facilitating consultation for diagnosis and treatment of RC and at promoting research. In the US, the National Clinical Trials Network launched in 2014 provides, with focus on RC and minorities, infrastructure for NCI‐funded treatment, screening, and diagnosis trials to improve the lives of patients with cancer (https://www.cancer.gov/research/areas/clinical‐trials/nctn).

Recently, the definition of cancer entities was revised^5^ according to the 4th version of the WHO classification of tumors publication, following the new WHO Blue Books (http://whobluebooks.iarc.fr/). This new definition was applied to the European data and the Surveillance, Epidemiology, and End Results (SEER) data.^6^

Although the two cited studies^1^, ^2^ reported similar general patterns of RC, they were not powered to identify specific differences in US and Europe because they were conducted independently using different definitions, diagnosis period, and methods. The aim of this paper was to use the revised list of RC and a common methodology to provide a more in‐depth comparison of incidence and survival for RC in the US vs Europe. These comparisons have the potential to highlight important differences and improve our understanding of RC in both locations.

## 2 MATERIAL AND METHODS

### 2.1 Cases

We analyzed incidence and survival data collected in 18 SEER registries^6^ and in 94 European registries included in the RARECAREnet website.^5^ Only population‐based registries were considered. They are aimed at collecting all the cases diagnosed in the reference population by merging all the available demographical, clinical, pathological, and laboratory information, and are bound to code data according to internationally agreed protocols. In addition, CRs follow incident cases for vital status, mainly by linking cancer registry database with the mortality database of the National Statistical Office. Cause of death is collected by SEER registries but not by all European registries. We included all malignant cancers diagnosed during the period 2000‐2007. This period was chosen because it is the most recent period for which European data are available. Different primary RC in a same patients were included in the analysis but only the first primary of a given tumor was considered.

### 2.2 Methods

Rare cancers entities were defined according to the updated RARECAREnet list (Table S1), slightly revised using topography and morphology codes from the third edition of the International Classification of Diseases for Oncology (ICD‐O‐3) (https://apps.who.int/iris/handle/) and the fourth version of the WHO classification of tumors (http://whobluebooks.iarc.fr/). Cancers that satisfied the RC definition criteria (ie crude incidence rates <6/100 000/y) in SEER and European data were identified, and only those that were rare in both datasets were selected to conduct more detailed comparisons.

US and European data were collected using common protocols worldwide agreed within the community of population based cancer registriestrs^7^, but they might differ according to the compliance to such protocols. As for data quality indicators, the percentage over all cases of those detected from death certificate only were 1.0% in US vs 1.6% in EU, while the proportion of RC censored alive with zero survival time was 0.2% in both datasets. Finally, the proportion of all cases with not otherwise specified (NOS) morphology codes was 3.2% and 9.6% for solid tumors (M8000‐8001, 8800‐8801), and 7.1% and 13.5% for hematological tumors (M 9590‐9591, 9760, 9800‐9801, 9820, 9860) in the US and Europe, respectively.

Incidence was calculated as the ratio between the number of all new cases diagnosed with a given tumor and the number of person‐years lived in the reference population during the considered period. Age‐adjusted incidence rates (ASR, USA 2000 standard population) were compared between SEER and EU in terms of absolute differences and using the *z*‐test for statistical significance.

We estimated cancer‐specific survival at 5‐year from diagnosis by the net survival (NS) indicator, based on the excess mortality from all causes of patients with respect to age and sex comparable general population groups. We calculated NS by the Pohar‐Perme method,^8^ the standard method to provide unbiased survival comparisons between populations subjected to different non‐cancer mortality risks. Survival comparisons used the absolute difference between US and EU NS.

Incidence and survival comparisons by age at diagnosis groups are provided using funnel plots,^9^ which are useful to visualize the distribution of a large number of estimates (here, large number of entities). For incidence, the dots represent the ratio (RR) between the ASR in the US divided by that in Europe, and the cone‐shaped area delimited RRs that are not significant by the 3‐standard deviations criterion, a threshold normally used^9^, ^10^ in funnel plot analysis of many statistical units, corresponding to 99.8% confidence limits. For survival, dots represent the difference of 5‐year NS in the US minus 5‐year NS in Europe, and are again represented together with the 3‐standard deviation confidence limits. All the analyses were carried out by SEER*Stat 4.0 software.

## 3 RESULTS

### 3.1 Incidence

**FIGURE 1:**
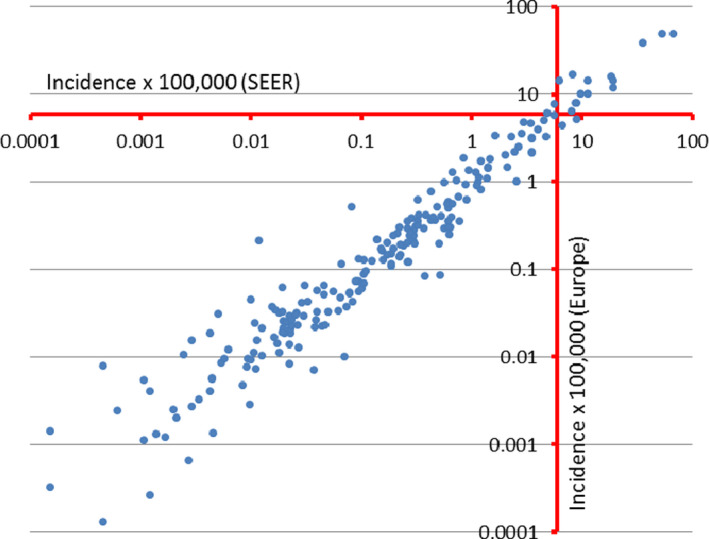
Crude annual incidence rates for cancer entities in Europe (*y*‐axis) and the US (*x*‐axis)

The crude annual incidence rate for all RC together was slightly but significantly lower in the US (114/100 000) than Europe (118/100 000) (Table 1). However, the European population was older (17% vs 22% aged 0‐14 and 16% vs 11% aged 65+), so the ranking reversed when comparing ASR (118 vs 101), with the US significantly higher than Europe. Age‐specific incidence rates were lower in EU than in US for the older age classes, so the age distribution of RC cases was similar between the two population. Rare and common cancers in the two populations are represented in Figure 1, which plots crude incidence rates estimated in Eutope (y‐axis) vs the US (*x*‐axis). According to the RC definition (crude incidence <6/100 000), two cancers (*thyroid carcinoma* and *diffuse B lymphoma*) classified as rare in the European population, however, were common in the US (Figure 1, low right square). In contrast, *breast invasive lobular carcinoma* was common in Europe and rare in the US (Figure 1, high left square). Table 1 shows that incidence of *thyroid carcinoma* and *diffuse B cell lymphoma* was, respectively, 80% and 55% higher in the US compared with Europe. *Breast invasive lobular carcinoma* had almost 40% lower incidence in the US than in Europe. From this point forward, we only considered entities that were rare in both datasets, and we excluded *thyroid carcinoma, diffuse B lymphoma* and *breast lobular carcinoma*.

**TABLE 1:** Comparison between rare cancers (RC) in Europe and the US (2000‐2007 diagnoses). US and European populations, RC proportions, and rate ratio (a). Number of incidence cases (N), incidence rates standard errors (SE) and incidence rate ratios (b); 5‐y net‐survivals (NS) and 5‐y absolute survival differences (c)

(a) Age and sex	SEER			EU			SEER/EU rate ratio
	Population (%)	Rate	RC cases (%)	Population (%)	Rate	RC cases (%)	
00‐14 y	22	13.9	3	17	11.8	2	1.18
15‐24 y	14	18.8	2	13	19.5	2	0.96
25‐44 y	29	48.2	12	29	49.0	12	0.98
45‐54 y	14	128.6	16	14	120.3	14	1.07
55‐64 y	9	241.1	20	11	213.2	21	1.13
65‐74 y	6	408.5	21	9	323.5	24	1.26
75+ y	5	541.9	26	7	408.8	25	1.33
Male	49	110.4	48	49	120.1	50	0.92
Female	51	117.6	52	51	114.9	50	1.02

(b) Incidence comparisons	Count	Rate	SE	Count	Rate	SE	SEER/EU rate ratio
All rare cancers (crude)	741 658	114.1	0.13	1 840 137	117.5	0.09	0.97
All rare cancers (stand)	741 658	118.3	0.14	1 840 137	100.7	0.07	1.17
Incidence discordances							
Invasive lobular carcinoma of breast^a^	36 623	5.63	0.03	121 455	7.75	0.02	0.73
Carcinoma of thyroid gland^b^	59 218	9.11	0.04	79 420	5.07	0.02	1.80
Diffuse B lymphoma^b^	43 414	6.68	0.03	67 645	4.32	0.02	1.55

(c) Survival comparisons	Count	5‐y surv (%)	SE (%)	Count	5‐y surv (%)	SE (%)	SEER‐EU 5‐y survival difference (%)
Cancers rare in each dataset	722 170	53.6	0.1	1 787 618	48.2	0.1	5.4
Invasive lobular carcinoma of breast^a^	36 044	90.8	0.3	120 973	85.8	0.1	5.0
Carcinoma of thyroid gland^b^	58 573	96.1	0.2	78 533	90.1	0.2	6.0
Diffuse B lymphoma^b^	43 199	56.3	0.3	67 907	52.6	0.3	3.7
Cancers rare in both datasets	686 274	51.6	0.1	1 643 618	46.0	0.1	5.6

Population and RC cases columns show age‐ and sex‐specific percentages of the total population and overall cases. Rates are per 100 000 and age‐adjusted to the USA 2000 standard population.

^a^ Common in EU.

^b^ Common in SEER.

Among a total of 196 entities rare in both countries, we reported in Table 2 those with incidence rates significantly different in the two populations by an (arbitrarily chosen) absolute difference of at least 0.2 per 100 000. Most of them (34 out of 43 entities) had higher incidence in the US compared to Europe. Lower incidence in the US compared to Europe was observed for nine entities, including *squamous cell carcinoma of larynx*, *esophagus*, and *cervix uteri*, and *esophageal adenocarcinoma* (Table 2). Also accounting for the small number of cases through the precision level, differences in incidence appear to be minor for tumors occurring in patients aged <25 years (Figures S1‐S5).

**TABLE 2:** ASR of rare cancers with ASR statistically different between Europe and the US, and absolute difference >0.2. Number of cases (N), standard error (SE), 5‐year net survival (NS), incidence and survival absolute difference between the European and the US, also reported

Cancers		US‐SEER			EUROPE			Incidence difference	Survival difference (%)
		N	ASR (SE)	NS (%)	N	ASR (SE)	NS (%)		
Head and neck	Epithelial tumor of major salivary glands	7550	1.21 (0.014)	69	15 067	0.82 (0.007)	60	0.384	9
	SCC of hypopharynx	4396	0.7 (0.011)	29	19 828	1.06 (0.008)	25	−0.358	4
	SCC of larynx	22 337	3.57 (0.024)	60	72 210	3.77 (0.014)	60	−0.209	−1
	SCC of oropharynx	22 816	3.55 (0.024)	56	49 004	2.65 (0.012)	41	0.895	15
Rare digestive	SCC of esophagus	10 801	1.75 (0.017)	14	52 597	2.74 (0.012)	11	−0.995	3
	Adenocarcinoma of esophagus	15 049	2.43 (0.02)	18	51 138	2.66 (0.012)	13	−0.238	5
	SCC anal canal	7993	1.26 (0.014)	68	12 691	0.69 (0.006)	62	0.569	6
	Hepatocellular carcinoma of liver and IBT	30 828	4.88 (0.028)	15	50 461	2.6 (0.012)	14	2.290	1
	Adenocarcinoma of extrahepatic biliary tract	9315	1.52 (0.016)	16	22 507	1.16 (0.008)	19	0.359	−3
Rare thoracic	Adenosquamous carcinoma of lung	3738	0.61 (0.01)	28	4607	0.24 (0.004)	22	0.376	6
	Large cell carcinoma of lung	13 178	2.15 (0.019)	14	31 589	1.63 (0.009)	10	0.513	4
	Mesothelioma of pleura and pericardium	5534	0.92 (0.012)	5	28 676	1.46 (0.009)	4	−0.544	0
Rare female genital	Special types of adenocarcinoma of breast	23 213	3.72 (0.025)	98	48 290	2.62 (0.012)	95	1.100	3
	Serous (papillary) carcinoma of corpus uteri	3427	0.56 (0.01)	45	1317	0.07 (0.002)	40	0.492	5
	Mullerian mixed tumor of corpus uteri	3509	0.57 (0.01)	38	6293	0.32 (0.004)	36	0.247	2
	SCC of cervix uteri	19 443	3.03 (0.022)	69	74 103	4.37 (0.016)	66	−1.338	2
	Mucinous adenocarcinoma of ovary	2820	0.44 (0.008)	57	12 066	0.67 (0.006)	60	−0.227	−3
	Primary peritoneal serous/papillary carcinoma of ovary	2478	0.4 (0.008)	30	1280	0.07 (0.002)	21	0.337	8
Rare male genital and urogenital	Infiltrating duct carcinoma of prostate	535	0.09 (0.004)	84	8064	0.4 (0.005)	78	−0.315	6
	Seminomatous testicular cancer	9730	1.49 (0.015)	98	28 516	1.81 (0.011)	97	−0.320	1
	Transitional cell carcinoma of pelvis and ureter	9186	1.52 (0.016)	48	21 975	1.13 (0.008)	51	0.395	−3
Rare skin	Adnexal carcinoma of skin	3969	0.64 (0.01)	91	5534	0.3 (0.004)	83	0.347	8
Sarcomas	STS of limbs	8983	1.42 (0.015)	74	17 186	0.96 (0.007)	67	0.459	7
	STS of superficial trunk	4075	0.65 (0.01)	55	7807	0.43 (0.005)	48	0.217	7
	STS of skin	4202	0.66 (0.01)	91	4734	0.28 (0.004)	90	0.389	1
	Gastrointestinal stromal sarcoma	4034	0.64 (0.01)	72	4706	0.25 (0.004)	72	0.394	0
	Kaposi s sarcoma	4115	0.65 (0.01)	67	3893	0.23 (0.004)	79	0.423	−12
Rare neuroendocrine	Well diff. not funct. endocrine carcinoma of GEP	16 683	2.62 (0.02)	84	15 852	0.86 (0.007)	71	1.763	13
	Poorly differentiated endocrine carcinoma	4984	0.8 (0.011)	33	10 421	0.55 (0.005)	35	0.244	−2
	Typical and atypical carcinoid of the lung	4321	0.69 (0.011)	87	6160	0.34 (0.004)	81	0.349	7
	Neuroendocrine carcinoma of skin	3312	0.55 (0.01)	54	3026	0.16 (0.003)	55	0.388	−1
	Neuroendocrine carcinoma of other sites	7265	1.17 (0.014)	24	14 120	0.75 (0.006)	24	0.427	1
CNS	Astrocytic tumors of CNS	29 997	4.75 (0.028)	18	78 005	4.31 (0.016)	15	0.444	3
Rare hemathologic	Hodgkin lymphoma, classical	17 333	2.67 (0.02)	81	38 588	2.37 (0.012)	81	0.305	0
	Precursor B/T lymphoblastic leuk/lymphoma	13 827	2.14 (0.018)	60	22 795	1.57 (0.011)	58	0.568	2
	T cutaneous lymphoma	5088	0.81 (0.011)	84	5526	0.3 (0.004)	81	0.506	3
	Other T cell lymphomas and NK cell neoplasms	5903	0.94 (0.012)	40	9656	0.53 (0.005)	39	0.406	1
	Plasmacytoma/multiple myeloma	36 867	5.99 (0.031)	38	89 440	4.65 (0.016)	34	1.340	4
	Mantle cell lymphoma	4407	0.71 (0.011)	50	8748	0.45 (0.005)	42	0.262	7
	Acute myeloid leukemia	26 247	4.25 (0.026)	16	60 891	3.33 (0.014)	17	0.919	0
	Chronic myeloid leukemia	7694	1.22 (0.014)	65	17 473	0.97 (0.007)	53	0.256	12
	Other myeloproliferative neoplasms	16 073	2.59 (0.021)	80	33 954	1.82 (0.01)	73	0.774	7
	Other myelodysplastic syndromes	22 887	3.8 (0.025)	40	33 542	1.79 (0.01)	31	2.011	9

Abbreviations: ASR, age‐adjusted incidence rates; GEP, gastroenteropancreatic tract; IBT, intrahepatic biliary tract; NS, net survival; SCC, squamous cell carcinoma; SE, standard error; STS, Soft tissue sarcoma.

### 3.2 Survival

**FIGURE 2:**
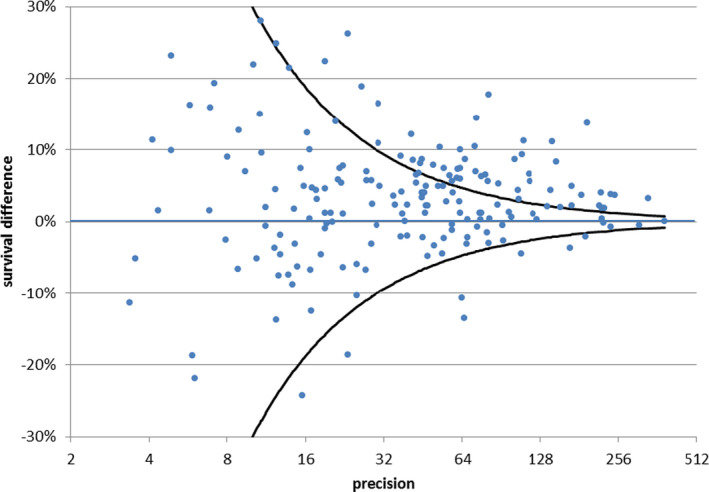
Difference between 5‐y net survival (NS) for rare cancers (RC) in US and Europe, period of follow‐up 2000‐2007. Funnel plot in which each dot represents a single RC, the *y*‐axis displays the estimated difference in 5‐y NS, and the *x*‐axis the corresponding precision in terms of the inverse of its SE. Three‐SE confidence bounds are represented by two symmetrical lines progressively approaching the *y* = 0 line with increasing *x* values. Dots lying above or below the area between them correspond respectively to tumors with 99.8% significantly higher or lower NS

Five‐year NS for all RC combined significantly differed: 54% in the US and 48% in Europe (Table 1). Figure 2 shows a funnel plot representing 5‐year NS differences in the US vs Europe and the 3‐standard deviations control limits for 187 entities with at least 5 cases in each population. For most entities, the dots representing NS lie between the 3‐standard errors confidence bounds. Survival was higher in the US for 62 and higher in Europe for 6 entities that fall respectively above and below the limits. Entities with differences in survival were not concentrated in a particular cancer family. Table 3 lists 47 outlier entities with estimated differences >5% in absolute value. *Prostate transitional carcinoma, sarcoma of Kaposi,* and *visceral sarcoma* had significantly better survival in Europe. However, most entities (44) had better survival in the US, with the most important differences (>15%) estimated for example for *head and neck salivary gland type tumor, lung salivary gland type tumor, chordoma, eye and adnexa adenocarcinoma, CNS oligodendroglial tumors,* and *histiocytic malignancies*.

**TABLE 3:** 5‐y net survival of rare cancers with net survival statistically different between Europe and the US. Number of cases (N), Net survival and corresponding standarad errors (SE), age standardized incidence rates (ASR), and absolute survival and incidence differences between Europe and the US, also reported

Cancers		US‐SEER			Europe			Survival difference (%)	Incidence difference
		N	Net Surv (SE)	ASR	N	Net Surv (SE)	ASR		
Head and neck	SCC of nasopharynx	3038	55.6 (1.12)	0.473	5589	48.1 (0.8)	0.316	8	0.157
	Epithelial tumor of major salivary glands	7493	69.3 (0.8)	1.206	14 717	60.1 (0.58)	0.822	9	0.384
	Salivary gland type tumor of head and neck	2962	82.2 (1.1)	0.471	6684	66.6 (0.81)	0.367	16	0.105
	SCC of oropharynx	22 571	55.6 (0.43)	3.548	48 584	41.1 (0.27)	2.653	15	0.895
	SCC of oral cavity	17 959	53.8 (0.52)	2.947	54 229	48 (0.28)	2.957	6	−0.011
Rare digestive	SCC of rectum	1209	59.6 (1.88)	0.192	1777	46.5 (1.54)	0.094	13	0.098
	SCC of anal canal	7954	67.9 (0.73)	1.258	12 847	62.2 (0.6)	0.688	6	0.569
	Intraductal papillary mucinous carcinoma of pancreas	119	57.6 (6.29)	0.019	171	31.4 (4.9)	0.009	26	0.011
Rare thoracix	Adenosquamous carcinoma of lung	3718	28.1 (0.97)	0.612	4566	21.8 (0.76)	0.236	6	0.376
	Salivary gland type tumor of lung	261	66.5 (3.84)	0.041	880	39.7 (1.94)	0.049	27	−0.007
Rare female genital	Metaplastic carcinoma of breast	1708	70.6 (1.69)	0.272	1897	63.6 (1.62)	0.103	7	0.170
	Adenocarcinoma of cervix uteri	5771	73.7 (0.72)	0.905	14 221	66.6 (0.47)	0.837	7	0.067
	Clear cell adenocarcinoma of ovary	2022	66.2 (1.31)	0.311	4761	55.5 (0.88)	0.258	11	0.054
	Primary peritoneal serous/papillary carcinoma of ovary	2474	29.7 (1.23)	0.402	1280	21.4 (1.55)	0.066	8	0.337
	Adenocarcinoma of falloppian tube	1296	68.4 (1.82)	0.207	2690	58.8 (1.26)	0.140	10	0.066
	Malignant/immature teratoma of ovary	508	93.6 (1.21)	0.077	829	83.1 (1.47)	0.053	11	0.024
	Germ cell tumor of ovary	620	92.6 (1.13)	0.094	1143	86.5 (1.12)	0.077	6	0.017
	SCC of vulva and vagina	7852	68.5 (0.8)	1.283	26 271	59.5 (0.48)	1.415	9	−0.133
	Adenocarcinoma of vulva and vagina	607	55.9 (2.65)	0.097	1115	45.9 (1.92)	0.060	10	0.037
Rare male genital and urogenital	Transitional cell carcinoma of prostate	125	33.8 (5.96)	0.021	941	56.6 (2.41)	0.049	−23	−0.028
Epithelial tumor of eye and adnexa	Adenocarcinoma of eye and adnexa	112	75.2 (5.76)	0.018	218	52.8 (4.33)	0.012	22	0.006
Mesothelioma	Mesothelioma of peritoneum and tunica vaginalis	618	21.1 (2.05)	0.100	1999	12.7 (0.93)	0.107	8	−0.007
Rare melanomas	Malignant melanoma of uvea	3194	82.3 (1.16)	0.508	8024	70.7 (0.77)	0.311	12	0.075
Rare skin	Adnexal carcinoma of skin	3864	91 (1.26)	0.643	5503	83.4 (1.25)	0.296	8	0.347
Embrional	Neuroblastoma and ganglioneuroblastoma	1441	73.8 (1.31)	0.220	2135	68 (1.09)	0.178	6	0.042
Sarcomas	STS of limbs	8934	73.7 (0.69)	1.420	17 101	67.1 (0.51)	0.961	7	0.459
	STS of superficial trunk	4056	54.9 (1.03)	0.647	7717	47.7 (0.73)	0.430	7	0.217
	STS of viscera	1845	32.1 (1.33)	0.299	5856	42.1 (0.8)	0.318	−10	−0.019
	STS of retroperitoneum and peritoneum	2127	45 (1.38)	0.342	4851	38.2 (0.86)	0.264	7	0.078
	STS of pelvis	2000	55.3 (1.44)	0.318	3008	47.3 (1.13)	0.169	8	0.149
	Ewing's sarcoma of soft tissue	690	54.1 (2.08)	0.106	1079	44.8 (1.69)	0.071	9	0.035
	Osteogenic sarcoma	1933	58.2 (1.28)	0.298	3757	50.6 (0.93)	0.249	8	0.048
	Chondrogenic sarcoma	1720	77.6 (1.35)	0.270	4521	69.4 (0.89)	0.263	8	0.007
	Notochordal sarcoma, chordoma	573	76.9 (2.59)	0.091	1127	59.3 (1.97)	0.064	18	0.027
	Kaposi's sarcoma	4055	67.1 (1.04)	0.649	3830	78.8 (1.12)	0.226	−12	0.423
Rare neuroendocrine	Well diff not functioning endocrine carcinoma of GEP	16 439	84 (0.47)	2.625	15 656	71.4 (0.51)	0.862	13	1.763
	Well diff functioning endocrine carcinoma of GEP	150	83.6 (4.33)	0.024	407	60.3 (2.97)	0.023	23	0.001
	Typical and atypical carcinoid of the lung	4274	87.4 (0.88)	0.689	6058	80.6 (0.69)	0.340	7	0.349
CNS	Oligodendroglial tumors of CNS	2909	69.7 (0.99)	0.451	6124	51.5 (0.74)	0.365	18	0.086
	Ependymal tumors of CNS	1591	82.1 (1.21)	0.246	3185	72.7 (0.91)	0.204	9	0.042
	Neuronal and mixed neuronal‐glial tumors	55	78 (6.13)	0.008	74	49.4 (6.86)	0.005	29	0.004
Rare hemathologic	Malignant meningiomas	889	64.7 (2.12)	0.146	3117	55.8 (1.14)	0.183	9	−0.036
	Mantle cell lymphoma	4389	49.9 (1.08)	0.715	8797	42.4 (0.73)	0.453	7	0.262
	Chronic myeloid leukemia	7560	65.3 (0.75)	1.223	16599	53.2 (0.5)	0.967	12	0.256
	Other myeloproliferative neoplasms	15 861	80 (0.58)	2.591	33 599	73.4 (0.4)	1.817	7	0.774
	Other myelodysplastic syndrome	22 437	40.1 (0.54)	3.797	32 576	30.7 (0.39)	1.786	9	2.011
	Histiocytic malignancies	211	81.6 (3.06)	0.033	645	62.5 (2.28)	0.043	19	−0.011

Abbreviations: CNS, central nervous system; GEP, gastroenteropancreatic tract; SCC, squamous cell carcinoma; SE, standard errors; STS, soft tissue sarcoma.

Five‐year NS differences by age are represented, vs precision of estimates, in funnel plots, (Figures S6‐S9). The number of significant differences increased by age group. *CNS oligodendroglial tumors* were the only entity with significant survival differences for childhood cancers (<15 years), and only four RC differed in the adolescent and young adult aged 15‐24 years. The highest number of survival differences significantly higher in the US than European population, was found for cases aged at diagnosis >65 years.

Tables 2 and 3 also report, for the entities with most relevant differences in incidence and survival, their respective estimates of survival and incidence. The same data are also shown in Tables S2 and S3 with European incidence and survival indicators disentangled by European geographical region (North, UK & Ireland, Center, South and East). Overall, no major relation was found between the two indicators for the entities selected in the two tables. Only 14 cancers are listed in both tables. *Kaposi sarcoma* had higher incidence (0.6 vs 0.2) and lower survival (67 vs 79) in the US compared to Europe. The other 13 entities had both quantities higher in the US. Particularly striking incidence differences were estimated for *well differentiated not functioning endocrine carcinoma of GEP* (2.6 vs 0.9) and for *other myelodysplastic syndromes* (3.8 vs 1.8), to which correspond a survival advantage of 13 and 9 percentage points, respectively.

## 4 DISCUSSION

This first comparative analysis shows that both incidence and survival of RC, diagnosed during the period 2000‐2007, significantly differed between Europe and the US. We have to remind that, while health care is under the competence of single European countries, policy on rare disease (and rare cancers) is coordinated by EU. Important programs have been introduced in Europe, such as European Joint Actions on rare cancers,^3^ the establishment of European Reference Networks, and of cross‐border care directive. ^4^Furthermore, a universalist public health system is common to all European countries. This paper was aimed at giving baseline comparison data between Europe as a whole and a big country as US, differently organized with respect to health care system, policy plans and with large health care disparities.

Age‐adjusted incidence for all RC cancers combined was significantly higher (+17%) in the US than in Europe and almost half of the analyzed RC had higher incidence rates in the US. Possible reasons for such differences include the distribution of risk factors, “artificial” factors such as overdiagnosis, differences in the ability to diagnose RC, and different registration practices and use of classification codes in the two populations. In Figure S10, we show the major risk factors associated with increased risks for those RC that had significant differences in age‐adjusted incidence between the two populations and listed in Table 2. Below, we highlight the most important associations and provide insights on the differences where possible.

Tobacco is associated with increased risk for most of the epithelial RC and leukemias.^11^, ^12^, ^13^, ^14^ Although smoking prevalence is now slightly higher in Europe than the US,^15^ the incidence of many epithelial cancers and of leukemia, is higher in the US compared to Europe. However, prevalence of smoking has reduced more rapidly in the US than Europe, starting from similar rates in 1980.

Alcohol consumption is mainly related to the epithelial tumors of head and neck, liver, and esophagus,^11^, ^16^ however the consumption was higher at the beginning of this century and remains slightly higher in many of the major European countries (the UK, Germany, France Italy and the Netherlands).^17^ Actually, rates for esophageal and all the head and neck cancers, except the oropharyngeal cancer, are higher in Europe than in the US.

The occurrence of RC of the cervix, stomach, liver, oropharynx, nasopharynx, anogenital sites, lymphomas, and Kaposi sarcoma is related to specific infectious agents.^11^, ^16^ A recent study showed that the attributable fractions of cancer cases related to the infections were slightly higher in Europe compared to the US.^18^

Obesity is more prevalent in the US. Obesity/overweight is a risk factor for the epithelial tumors of the esophagus and extrahepatic biliary tract (EBT). Also, a fraction of pancreas, liver, kidney, corpus uteri, breast, ovary, and colorectal cancers are related to obesity, so rare entities of these cancer sites are likely associated with obesity as well.^16^

According to the International Agency for Research on Cancer (IARC) monographs and a review by Charbothel,^19^ many RC are consistently linked to occupational factors. However, it is difficult to estimate the prevalence of individuals exposed to occupational risk factors.

Access to effective screening, which is mostly organized in Europe and opportunistic in the US, can affect the incidence of cervix cancer and RC of the breast, colon and rectum. Screening initially increases the number of cases that are at an early stage at presentation and have high survival, and it subsequently leads to a decrease in incidence, perhaps followed by a stabilization. A different intensity of investigations for skin, kidney, prostate, and lung cancers could also affect the incidence of rare lesions in these cancer sites. Overdiagnosis leads to increased incidence and survival.

The incidence of *gastrointestinal stromal tumor (GIST), large cell carcinoma of lung*, neuroendocrine tumors, and many hematologic malignancies may be influenced by sounder pathological diagnosis and/or prompter and more correct adoption by registries of new entity codes in the ICD‐O‐3 coding procedures. We also know that the availability of tests for a correct diagnosis may be less in some European countries, such as in eastern Europe.^2^ Furthermore, a center of expertise with the option of a pathologic second opinion may be less available in some small European countries. Most tumors with higher incidence in the US than in Europe have recently been subject to classification changes; therefore, we can infer that part of the difference is explained by better tumor classification in the US by both pathologists and registrars.

Five‐year NS for all RC together was higher in the US than Europe. Many of the factors described above for incidence may also explain variations in survival,^16^ since different risk factors can generate biologically different diseases with different prognoses. This is the case for epithelial oropharyngeal cancer and squamous cell carcinoma of anogenital sites; a different proportion of tumors caused by HPV, characterized by less aggressive lesions, may have influenced the survival gap. A 2014 study showed that the proportion of HPV‐positive oropharynx cancers is higher in the US than in the European populations.^20^ The high prevalence of HIV‐infected individuals in the US, related to high AIDS rates, may explain the worse outcomes for the more aggressive form of Kaposi sarcoma in the US.^21^

Early stage at presentation increases therapy effectiveness and consequently improves survival. Timely access to new drugs (eg for chronic myeloid leukemia and other hematological malignancies), updated treatment protocols, and multimodality treatment influence survival. All these factors may be differentially available, creating disparities between countries. Although data are lacking on the patterns of care for the two populations and among the European countries, they may explain the higher survival for leukemia, especially CML, in the US compared to Europe. Overall, the organization of management of RC (centralization, hospital volume, second opinion, multidisciplinary approach, clinical research, network, etc) is crucial for improving the outcome, especially for patients with RC. Also, new diagnostic approaches relevant for improving problematic diagnoses, as those for many RC, can be differently available in the two regions, as well as the availability of more effective and expensive treatment protocols.

Incidence and survival are higher in the US than Europe^22^, ^23^ not only for rare but also for common cancers. The two regions also differ in health system organization: universalistic in Europe vs private in the US, and we cannot exclude the possibility that more intensive investigations in the US population produce higher cancer incidence and higher incidence of early‐stage cases, leading to a more favorable outcome. Furthermore, investments, management, and health care plans are different between European countries, even with universalistic provision of care. In all EUROCARE studies, age‐adjusted 5‐year survival varied markedly between Nordic and Central, and Eastern countries; this has also been shown for RC combined, even after adjusting by case mix.^24^ Eastern European countries had lower survival, and also lower incidence, for many RC (Tables S2 and S3), but they only contributed by 15% of the considered cases and 17% of the total amount of person‐years. Removal of Eastern European countries only slightly changed (not shown in tables) the incidence and survival differences between EU and US.

The two datasets considered in this analysis have been consistently used in previous comparative population based international studies.^22^, ^23^ Such studies were, however, mainly addressing common cancers. A reliable epidemiological description of RC entities requires a low proportion of cases detected by death certificates and of cases with NOS morphology, as different and high proportions of unknown morphology codes can affect the quality of comparison. In our study, the proportions of DCO was negligible in both datasets, while those of NOS were higher in Europe than in US. A SEER based study has shown that breast cancers with missing information on biomarkers had poorer prognosis than those with reported information.^25^ It may be plausible that tumors coded as NOS are more likely to be rare or of more complex diagnosis and poor prognosis. Thus, a higher proportion of NOS morphologic groups is indicative of incidence underestimation for some RC.

The European database includes 94 registries, and even with great standardization efforts, thanks to the European Network of Cancer Registries (ENCR), and to centralized quality checks by the EUROCARE and RARECAREnet groups, one cannot eliminate a certain level of heterogeneity in registration among the European registries, which may be greater than in the 18 US registries considered.

The study period considered in this paper is not recent, because of challenges in obtaining more updated data from the European registries. However, by using the same period of diagnosis, we provide more accurate comparisons than previous papers.^1^, ^2^ Finally, clinical information on stage and treatment is incompletely collected by many European registries, which precludes a more extensive comparison.

Centralization and networking, the most appropriate answers to the issues pertaining to RC, were not optimal in Europe during the period of our analisys,^2^ but we know about ameliorative efforts in the national organization plans, in the majority of the studied countries.^2^ An opportunity to improve survival (and possibly reduce incidence) in Europe, however too recent to have impacted on the results of this paper, comes from the implementation of the ERNs. The Joint Action of Rare Cancers, the major European initiative aimed at prioritizing RC in the agendas of the European Member States, ended in 2019.

The role of population based cancer registries still remains crucial to describe the real world impact of cancer and to evaluate progresses made. Their results have to be as much as possible uniformed at the European level in timeliness and in quality. This study suggests an important role for registration/classification practices, which should be enabled to keep up with advances in cancer research, codifying with more precision the cancer's topography, morphology, stage, and possibly other prognostic factors. We benefited from the RARECARE definition of RC. The corresponding list will be made available soon, and a variable in the SEER*Stat software will provide the means to conduct further studies on RC entities in the US.

In conclusion, we have shown using standardized methods that differences exist on incidence and survival of RC in the US and Europe. We provided some interpretation for incidence and survival differences. Our findings suggest opportunities for further research to understand the burden of RC and to spur greater international collaboration on the study of RC, with the goal of greater awareness, knowledge and therefore providing inputs in their prevention, diagnosis and treatment. We, also, provided basic information for the organization of clinical studies. In RC research, the larger is the collaboration the greater the chance to make progress.

## CONFLICT OF INTEREST

None.

## AUTHOR CONTRIBUTIONS

Laura Botta contributed to conceptualization, data curation, formal analysis, methodology, software, visualization, writing – original draft and writing – review and editing. Riccardo Capocaccia contributed to conceptualization, data curation, formal analysis, methodology, funding acquisition, software, writing – original draft, and writing – review and editing. Annalisa Trama contributed to data curation, project administration, and writing – review and editing. Alice Bernasconi contributed to data curation and writing – review and editing. Elad Sharon contributed to writing – review and editing. Gemma Gatta contributed to conceptualization, data curation, methodology, funding acquisition, project administration, writing – original draft, and writing – review and editing. Angela Mariotto contributed to conceptualization, methodology, funding acquisition, project administration, writing – original draft, and writing – review and editing.

## Supporting information

Supplementary Material

Supplementary Material

## Data Availability

The US data that support the findings of this study are openly available in “SEER” database at www.seer.cancer.gov. The European data that support the findings of this study are available on request from the corresponding author, conditional to the agreement of the contributing cancer registries. The data are not publicly available due to privacy restrictions.
