# Supplementary material for: Incidence and survival of rare cancers in the US and Europe
Source: Cancer Med. 2020 May 21;9(15):5632–42. doi: 10.1002/cam4.3137 (PMC7402819; doi:10.1002/cam4.3137)
Supplement: Supplementary file 2 — Supplementary Material [file CAM4-9-5632-s002.docx]

**RARECARENet Working Group:**

Monika Hackl (Austrian National Cancer Registry); Elizabeth Van Eycken, Kris Henau (Belgian Cancer Registry); Nadya Dimitrova, Zdravka Valerianova (Bulgaria Cancer Registry); Mario Sekerija (Croatian Cancer Registry); Ladislav Dušek (Czech National Cancer Registry); Margit Mägi (Estonian Cancer Registry); Keiu Paapsi (National Institute for Health Develpoment, Estonia); Nea Malila, Maarit Leinonen (Finnish Cancer Registry); Michel Velten (Bas Rhin Cancer Registry); Xavier Troussard (Basse Normandie Haematological malignancies Registry); Veronique Bouvier (Calvados Digestive Tract Registry); Anne-Valérie Guizard (Calvados, Registre Gèneral des Tumeurs); Anne-Marie Bouvier (Côte d'Or Digestive Tract Registry, Burgundy); Patrick Arveux (Côte d'Or Gynaecologic Cancer registry); Marc Maynadié (Côte d'Or Haematological Malignancies Cancer Registry); Anne-Sophie Woronoff (Doubs Cancer Registry); Michel Robaszkiewicz (Finistère Digestive Tract Registry); Isabelle Baldi (Gironde CNS Tumour Registry); Alain Monnereau (Gironde Haematological Malignancies); Brigitte Tretarre (Hérault Cancer Registry); Marc Colonna (Isère Cancer Registry); Florence Molinié, Anne Cowppli-Bony (Loire-Atlantique/Vendée Cancer Registry); Simona Bara (Manche Cancer Registry); Claire Schvartz (Marne & Ardennes, Thyroid); Bénédicte Lapôtre-Ledoux (Somme Cancer Registry); Pascale Grosclaude (Tarn Cancer Registry); Roland Stabenow (Berlin; Brandenburg; Mecklenburg-West Pomerania; Saxony; Saxony-Anhalt; Thüringen Cancer Registry); Sabine Luttmann (Bremen Cancer Registry, Leibniz Institute for Prevention Research and Epidemiology); Alice Nennecke (Hamburg Cancer Registry); Jutta Engel, Gabriele Schubert-Fritschle (Munich Cancer Registry); Jan Heidrich (North Rhine-Westphalia Cancer Registry); Bernd Holleczek (Saarland Cancer Registry); Jón Gunnlaugur Jónasson, Helgi Birgisson (Icelandic Cancer Registry); Kerri Clough-Gorr, Harry Comber (National Cancer Registry Ireland); Guido Mazzoleni (Alto Adige Cancer Registry); Adriano Giacomin (Biella Cancer Registry); Antonella Sutera Sardo (Catanzaro Cancer Registry); Alessandro Barchielli (Firenze-Prato Cancer Registry); Diego Serraino, Paolo Collarile (Friuli Venezia Giulia, CRO Aviano National Cancer Institute); Roberta De Angelis, Silvia Francisci, Corrado Di Benedetto, Sandra Mallone, Daniela Pierannunzio, Andrea Tavilla, Silvia Rossi, Mariano Santaquilani (Istituto Superiore di Sanità, Rome); Fabio Pannozzo (Latina Cancer Registry); Paolo Ricci (Mantova Cancer Registry); Mariangela Autelitano, Antonio Giampiero Russo (Milano Cancer Registry); Gianbattista Spagnoli (Modena Cancer Registry); Mario Fusco (Napoli 3 South Cancer Registry); Mario Usala (Nuoro Cancer Registry); Francesco Vitale (Palermo Cancer Registry); Maria Michiara (Parma Cancer Registry); Rosario Tumino (Ragusa Cancer Registry); Lucia Mangone (Reggio Emilia Cancer Registry); Fabio Falcini (Romagna Cancer Registry, Istituto Scientifico Romagnolo per lo Studio e la Cura dei Tumori (IRST), IRCCS, Meldola, Forlì, Italy-Azienda Usl della Romagna, Forlì, Italy); Rosa Vattiato (Romagna Cancer Registry); Stefano Ferretti (Ferrara Cancer Registry); Rosa Angela Filiberti, Enza Marani (RTR Liguria, IRCCS AOU SM-IST); Arturo Iannelli, Anna Luisa Caiazzo (Salerno Cancer Registry); Rosaria Cesaraccio(Sassari Cancer Registry); Silvano Piffer, Maria Gentilini (Servizio Epidemiologia Clinica e Valutativa, Trento); Anselmo Madeddu, Antonino Ziino Colanino (Siracusa Cancer Registry); Sergio Maspero (Sondrio Cancer Registry); Pina Candela (Trapani Cancer Registry); Fabrizio Stracci (Umbria Cancer Registry); Giovanna Tagliabue (Varese Province Cancer Registry, Fondazione IRCCS Istituto Nazionale dei Tumori); Massimo Rugge (Azienda Zero, SER - Registro Tumori del Veneto); Paolo Baili, Laura Botta, Riccardo Capocaccia, Roberto Foschi, Gemma Gatta,Pamela Minicozzi, Annalisa Trama, Carmen Tereanu, Milena Sant (Fondazione IRCCS, National Cancer Institute, Milan); Santa Pildava (Latvian Cancer Registry); Giedre Smailyte (Lithuanian Cancer Registry); Neville Calleja, Dominic Agius (Malta National Cancer Registry, Health Information and Research); Tom Børge Johannesen (Norwegian Cancer Registry); Jadwiga Rachtan (Cracow Cancer Registry); Stanisław Góźdź (Kielce Cancer Registry); Jerzy Błaszczyk, Kamila Kępska (Lower Silesia Cancer Registry, Wroclaw); Gonçalo Forjaz de Lacerda (Açores Cancer Registry); Maria José Bento (Northern Portugal Cancer Registry); Ana Miranda (Southern Portugal Cancer Registry); Chakameh Safaei Diba (Slovakian National Cancer Registry); Maja Primic-Žakelj, Tina Žagar (Cancer Registry of Republic of Slovenia); Enrique Almar (Albacete Castilla-La Mancha Cancer Registry); Nerea Larrañaga, Arantza Lopez de Munain (Basque Country Cancer Registry); Ana Torrella-Ramos (Castellón-Valencia (breast) Cancer Registry); José María Díaz García (Cuenca Cancer Registry); Rafael Marcos-Gragera (Girona Cancer Registry); Maria Josè Sanchez (Granada Cancer Registry , CIBERESP, ibs.Granada); Mª Dolores Chirlaque, Diego Salmerón (Murcia Cancer Registry, CIBERESP, IMIB-Arrixaca, Murcia University); Eva Ardanaz, Marcela Guevara (Navarra Cancer Registry, CIBERESP); Jaume Galceran, Marià Carulla (Tarragona Cancer Registry); Mohsen Mousavi (Basel Cancer Registry); Christine Bouchardy (Geneva Cancer Registry); Silvia M. Ess (Grisons-Glarus, St. Gallen Cancer Registry); Andrea Bordoni (Ticino Cancer Registry); Isabelle Konzelmann (Valais Cancer Registry); Jem Rashbass (Public Health England); Anna Gavin (Northern Ireland Cancer Registry); David H Brewster (Scotland Cancer Registry); Dyfed Wyn Huws (Welsh Cancer Intelligence and Surveillance Unit); Otto Visser, Jan Maarten van der Zwan, Sabine Siesling (The Netherlands Cancer Registry); Ellen Benhamou (Institut de Cancérologie Gustave Roussy, Villejuif, France); Renèe Otter (Scientific Institute of Public Health, Brussels)
